# The prevalence of smoking, second-hand smoke exposure, and knowledge of the health hazards of smoking among internal migrants in 12 provinces in China: a cross-sectional analysis

**DOI:** 10.1186/s12889-018-5549-8

**Published:** 2018-05-24

**Authors:** Yunting Zheng, Ying Ji, Hongbo Dong, Chun Chang

**Affiliations:** 1 Department of Social Medicine and Health Education, School of Public Health, Peking University, Beijing, 100191 People’s Republic of China; 2 Department of Personnel, Beijing University of Chinese Medicine, Beijing, 100029 People’s Republic of China; 3 Graduate School, Peking Union Medical College, Beijing, 100730 People’s Republic of China

**Keywords:** Smoking, Second-hand smoke, Internal migrants

## Abstract

**Background:**

Previous studies have provided inconsistent findings on smoking among migrants, and very limited data exist on their second-hand smoke exposure. This study aims to investigate internal migrants’ smoking prevalence, second-hand smoke exposure among non-smokers, and knowledge of the health hazards of smoking in 12 major migrant provinces in China in 2013.

**Methods:**

Data from the 2013 Migrant Dynamics Monitoring Survey in China published by the National Commission of Health and Family Planning was used in this study. Descriptive analysis, Chi-square analysis, and sex-stratified multivariate logistic regression analysis were used to explore the determinants of current smoking and second-hand smoke exposure.

**Results:**

Among 7200 migrants, 34.1% (55% male, 4% female) were current smokers. For males, factors associated with current smoking were education year (aOR = 0.95, 95% CI: 0.93–0.98), duration of stay (aOR = 1.01, 95% CI: 1.00–1.03) and occupation (aOR = 1.25, 95% CI: 1.03–1.53). For females, household registration status (aOR = 1.70, 95% CI: 1.04–2.80) was the most important factor associated with current smoking. Sixty five percent of non-smokers were exposed to second-hand smoke. Factors associated with exposure to second-hand smoke were duration of stay (aOR = 1.01, 95% CI: 1.00–1.02), divorced/widowed marital status (aOR = 0.48, 95% CI: 0.25–0.91), occupation (aOR = 1.29, 95% CI: 1.05–1.58) and the nature of employer (aOR = 0.77, 95% CI: 0.60–0.97). About 95% of participants were aware that lung cancer is one of the hazards of smoking. Non-current smokers had a better knowledge of fertility reduction and accelerated aging as hazards of smoking than current smokers (*p* < 0.01). Knowledge of the impact of smoking on cardiovascular diseases was relatively low compared with knowledge of other smoking-related hazards (26.1–44.3%).

**Conclusions:**

Current smoking and exposure to second-hand smoke among internal migrants in China is high. Socio-demographic characteristics and migration status were strongly associated with current smoking and second-hand smoke exposure. We recommend specifically targeted tobacco control interventions to help to address these risk factors, such as focusing on divorced/widowed women.

## Background

China is the world’s largest consumer of tobacco products and contributes substantially to the global burden of smoking-related diseases [1]. According to the Global Adult Tobacco Survey (GATS) conducted in 2010, 28.1% of adults in China were current smokers, with 72.4% of non-current smoking adults were exposed to second-hand smoke (SHS) in a typical week [2].

Internal migrants are defined as, ‘individuals who move within the borders of a country, usually measured across regional, district, or municipal boundaries, resulting in a change of usual place of residence’ [3]. The word ‘internal’ is used to differentiate this population from the cross-border migrants (generally called international migrants):‘individuals who remain outside their usual country of residence for at least one year’ [4]. China has a unique household registration system which functions as an ‘internal passport system’ to restrict Chinese citizens’ access to public services to their place of birth. Few can obtain local residency rights when they move away from their place of birth. This creates a two-system category of internal migrants: household registered and non-household registered, with the vast majority of internal migrants belonging to the latter.

In 2012 there were 236 million, or one in six, internal migrants in China [5], most of whom moved from less to more developed provinces and from villages to cities and towns. Most migrants are poorly educated, young and middle-aged men who take up low paying and labour-intensive jobs in the informal sector [6]. In addition, they have limited access to health services. Several studies have shown that migrants are at higher risk of communicable and non-communicable diseases, occupation-related conditions, sexual health problems, and psychological problems [7–9]. The stress induced by migration, and poor living and working conditions, are likely to increase the risk of substance abuse in this population [10].

Results of previous studies on smoking among internal Chinese migrants have been inconsistent: whilst some report a higher smoking prevalence among internal migrants than among urban residents [11, 12] others show the converse [13–15]. The same inconsistency is found in studies comparing international migrants to natives [16, 17]. A systematic review conducted by Liu et al. showed that smoking prevalence among male migrants was lower than the general population (pooled estimate 46.7% versus 52.9%) whilst smoking prevalence among female migrants was higher than the general population (5.3% versus 2.4%) [18]. However, a national study by Ji et al.*,* not included in this review [14], reported a lower smoking rate among migrants than the general population. The foregoing uncertainty around smoking in migrant populations is justification for a more thorough investigation of the subject.

There is a paucity of data from China on SHS exposure. One study reported that 43.6% of female internal migrants in China were exposed to SHS [19]. With regards to knowledge of the effects of smoking on health, a study on rural-to-urban migrant women in Beijing found that the proportion of migrant women who believed smoking increases the risk of cardiovascular disease, lung cancer and hepatitis was 61, 92, and 57% respectively [20]. Our literature search found no evidence that knowledge of other health hazards of smoking has been explored in this population.

This study aims to investigate smoking prevalence, exposure to SHS, and knowledge of the hazards of smoking and their determinants among internal migrants in 12 major migrant provinces in China in 2013.

## Methods

### Data sources

The current study is a secondary data analysis. We obtained data from the 2013 Migrant Dynamics Monitoring Survey in China. The survey was published by the National Population and Family Planning Commission and focused on basic public health services, health, and family planning for the Chinese migrant population. Respondents to the survey included men and women aged 15–59 years as of August 2013 who had been residing in a locality for six months or longer, with a non-local district (province, municipality, county) household registration. The survey used the annual national migrant reported data in 2012 as the basic information and adopted a four-stage sampling method.

In stage one, 12 of the 31 provinces of China were selected. These provinces included six that receive the highest number of internal migrants: Guangdong, Zhejiang, Beijing, Shanghai, Jiangsu and Fujian [21]. The others were Tianjin, Liaoning and Shandong (all eastern), Henan (central), and Chongqing and Sichuan (both western). In stage two, eight sub-districts in each province were randomly selected by PPS method. The sub-district sampling frame was obtained from the same national survey on migrant populations conducted by the National Population and Family Planning Commission in 2012 [22]. In stage three, a quota-sampling procedure was applied to recruit the participants. Work units were selected according to location, level of industrialization and topography. In stage four, eligible participants were selected at the sampling units. The number of participants in each sampling unit was limited to 15. Data collectors were drawn from the host communities. The enumerators contacted respondents by phone to explain the purpose of the study and request an appointment. Respondents who agreed to be interviewed were then visited and formal informed consent was obtained for interview. No identification information on survey participants was included in this study. The ethics approval was exempted by Peking University Institution Review Board. All participants were informed of the voluntary nature of the study, and provided verbal consent [23].

### Measures

We measured three outcomes: current smoking prevalence, SHS exposure, and knowledge of the hazards of smoking. The survey used the World Health Organization (WHO) global standard for smoking [24], where those responding “Yes” to “Do you currently smoke tobacco?” were considered current smokers. Nonsmokers were asked “How many days per week are you exposed to second-hand smoke usually?” Those who answered “none” were categorized as non-exposed to SHS and all others (“1-3 days per week”, “4-6 days per week” and “almost every day”) were categorized as exposed to SHS. All participants answered multiple choice questions on their knowledge of hazards of smoking: “Which of the following diseases do you think smoking can lead to?” Options included lung cancer, fertility reduction, accelerated aging, cardiovascular diseases, heart disease, and stroke. Participants answering “Yes” to any disease were deemed to have correct knowledge of the hazards of smoking.

Independent variables included basic demographic features (sex, age, marriage, years of education, and household registration type [agricultural and nonagricultural]), migration characteristics (migration type [trans-provincial, trans-municipal and trans-county], duration of stay [years], employment characteristics (employment status [yes/no], nature of employment [within the system,^1^ outside the system,^2^ agricultural, unemployed and others], and working hours per week.

### Statistical analysis

All statistical analyses were performed using SPSS13©. The sample was described using frequency counts and percentages for categorical variables and means and standard deviations (SD) for continuous variables. Chi-squared tests were performed to examine the associations between smoking prevalence and socio-demographic characteristics, migration history and working conditions. Differences in the knowledge of the hazards of smoking between current and non-current smokers were also tested with Chi-squared tests. Multivariate logistic regression was applied to identify independent variables associated with current cigarette smoking and with SHS, from which adjusted odds ratios (aOR) and 95% confidence intervals (95% CIs) were calculated. Smoking-related factors, including socio-demographic characteristics, employment and migration history were cross-tabulated with smoking status and SHS status. A *p*-value of less than 0.05 (two-tailed) was considered statistically significant. Due to the large sex differences in smoking, the regression model was sex-stratified.

## Results

### Characteristics of the study population

The survey sampled 7208 individuals and achieved a response rate 99.9%. General characteristics of the sample are shown in Table 1. Respondents ranged from 15 to 59 years of age, with a mean (SD) age of 33.3 ± 0.1. More men (59.9%) than women (40.1%) participated in the study (Table 1). The majority of respondents were married (82.5%) with agricultural household registration status (79.8%). The average number of years of education was 10.1 ± 0.4 years. More than 56% of participants were employed. The majority of migrants worked as business and service personnel (63.9%) and outside the system (81.6%). More than 60% of participants’ migrations were between-provinces.

**Table 1:** General characteristics of respondents

Variable	Male (*N* = 4313)		Female (*N* = 2887)		Total (*N* = 7200)	
	N	%	N	%	N	%
Age (m ± SD)(y)	33.89 ± 8.56		32.37 ± 8.46		33.28 ± 0.10	
Education year (m ± SD)(y)	10.24 ± 2.91		9.87 ± 3.30		10.09 ± 0.36	
Duration of stay (m ± SD)(y)	10.32 ± 6.64		8.84 ± 5.98		9.72 ± 6.43	
Working hours per week (m ± SD)(hrs.)	60.54 ± 17.75		57.42 ± 17.43		59.28 ± 17.69	
Marital status						
Never married	628	14.6	497	17.2	1, 194	16.6
Married	2, 625	60.9	2, 357	81.6	5, 937	82.5
Divorced/widowed	959	22.2	33	1.1	69	1.0
Household registration status						
Agricultural	3, 433	79.6	2, 309	80.0	5, 742	79.8
Nonagricultural	879	20.4	576	20.0	1, 458	20.2
Employment						
Yes	2, 437	56.5	1, 718	59.5	4, 155	57.7
No	1, 876	43.5	1, 169	40.5	3, 045	42.3
Occupational type						
Professional and technical personnel	628	14.6	238	8.2	866	12.0
Business and service personnel	2, 625	60.9	1, 976	68.4	4, 601	63.9
Production, transportation and facility operation personnel	959	22.2	490	17.0	1, 449	20.1
Agricultural, unemployed and others	101	2.3	183	6.3	1, 449	3.9
Employer nature						
Within the system	437	10.1	195	6.8	632	8.8
Outside the system	3, 478	80.6	2, 400	83.1	5, 878	81.6
Agricultural, unemployed and others	398	9.2	292	10.1	690	9.6
Migration type						
Trans-provincial	2, 835	65.7	1, 888	65.4	4, 723	65.6
Trans-municipal	1, 115	25.9	771	26.7	1, 886	26.2
Trans-counties	363	8.4	228	7.9	591	8.2

### Prevalence of smoking

Among the 7200 participants, 34.1% (95% CI: 33.0–35.2%) were current smokers; this is broken down by socio-demographic characteristics in Table 2. Current smoking was significantly higher among men than among women (54.5% vs. 3.7%, *p* < 0.001).

**Table 2:** Smoking prevalence and second-hand smoke exposure by demographics, migration history and working conditions

	Current smokers						SHS		
	Male			Female			Total		
Variables	N (%)	χ^2^	*P*	N (%)	χ^2^	*P*	N (%)	χ^2^	*P*
Age (y)									
15–29	780(53.0)	11.413	0.003	40(3.2)	6.447	0.040	1, 236(65.0)	0.026	0.987
30–39	924(53.1)			33(3.2)			1, 179(65.2)		
40–59	648(58.9)			33(5.4)			674(65.3)		
Education level									
Primary school and below	460(58.8)	39.688	< 0.001	29(4.3)	4.378	0.223	633(65.1)	0.235	0.972
Junior high school	1, 135(56.8)			39(3.1)			1, 338(64.8)		
Senior high school	513(53.0)			28(4.7)			674(65.7)		
College	244(43.3)			10(2.7)			444(65.3)		
Marital status									
Never married	372(53.4)	1.675	0.433	17(3.4)	12.462	0.002	544(67.7)	5.988	0.050
Married	1, 957(54.7)			84(3.6)			2, 524(64.8)		
Divorced/widowed	23(63.9)			5(15.2)			21(51.2)		
Duration of stay (y)									
0–2	120(74.5)	8.673	0.034	8(2.6)	2.507	0.474	326(71.3)	12.081	0.007
2–5	218(68.1)			24(3.9)			613(66.9)		
5–10	363(64.8)			37(4.4)			885(64.5)		
> 10	586(63.4)			37(3.3)			1, 265(63.3)		
Working hours per week (hrs.)									
≤ 40	427(50.0)	10.934	0.004	20(2.9)	1.768	0.413	673(60.8)	21.147	< 0.001
40–70	1, 434(56.4)			66(3.9)			1, 860(67.8)		
> 70	491(53.6)			20(4.1)			556(62.2)		
Household registration status									
Agricultural	1, 908(55.6)	7.573	0.003	75(3.2)	5.931	0.015	2, 437(64.8)	0.893	0.182
Nonagricultural	443(50.4)			31(5.4)			651(66.4)		
Employment									
Yes	1, 291(53.0)	5.484	0.019	60(3.5)	0.385	0.535	1, 838(65.6)	0.562	0.454
No	1, 061(56.6)			46(3.9)			1, 251(64.5)		
Occupational type									
Professional and technical personnel	283(45.0)	30.611	< 0.001	12(5.0)	3.715	0.294	350(61.3)	7.741	0.052
Business and service personnel	1, 449(55.2)			75(3.8)			2, 042(66.3)		
Production, transportation and facility operation personnel	565(58.9)			16(3.3)			546(63.0)		
Agricultural, unemployed and others	55(54.5)			3(1.6)			151(66.8)		
Employer nature									
Within the system	226(51.7)	5.503	0.064	11(5.6)	2.478	0.290	276(70.1)	10.858	0.004
Outside the system	1, 889(54.3)			86(3.6)			2, 549(65.3)		
Agricultural, unemployed and others	237(59.5)			9(3.1)			264(59.3)		
Migration type									
Trans-provincial	1, 558(55.0)	9.796	0.007	71(3.8)	1.58	0.454	1, 991(64.4)	12.594	0.002
Trans-municipal	574(52.5)			24(3.1)			832(64.6)		
Trans-county	220(60.6)			11(4.8)			266(73.7)		
Total	2, 352(54.5)			106(3.7)			3, 089(65.1)

**Table 3:** Knowledge of hazards of smoking

Knowledge of hazards of smoking	Current smokers (n, %)	Non-current smokers (n, %)	χ^2^	*P*
Lung cancer				
Yes	2, 335(95.0)	4, 493(94.7)	0.201	0.654
No	123(5.0)	249(5.1)		
Fertility reduction				
Yes	1, 260(51.3)	2, 660(56.1)	15.248	< 0.001
No	1, 198(48.7)	2, 082(43.9)		
Accelerated aging				
Yes	1, 215(49.4)	2, 656(56.0)	28.194	< 0.001
No	1, 243(50.1)	2, 086(44.0)		
Cardiovascular diseases				
Yes	1, 054(42.9)	2, 102(44.3)	1.377	0.241
No	1, 404(57.1)	2, 640(55.7)		
Heart disease				
Yes	839(34.1)	1, 684(35.5)	1.352	0.245
No	1, 619(65.9)	3, 058(64.5)		
Stroke				
Yes	641(26.1)	1, 248(26.3)	0.048	0.826
No	1, 817(73.9)	3, 494(73.7)		
Total				
Yes	398(16.6)	784(16.9)	0.114	0.735
No	1, 998(83.4)	3, 847(83.1)		

For men, the prevalence of current smoking was significantly higher among older respondents, respondents with less educational attainment, shorter duration of stay, working 40–70 h per week, with agricultural household registration status, with unemployment, and trans-county migration type. There were also some differences by occupational type and employer nature.

Women only shared one socio-demographic pattern as men: higher prevalence of current smoking among older respondents. Differences to men include a higher prevalence of current smoking among divorcees, and non-agricultural household registration status. Other socio-demographic characteristics did not significantly differ among women.

### Prevalence of SHS exposure

In total, 65.1% of non-smokers were exposed to SHS (95% CI: 64.0–66.2%) and there was no significant differences between males and females (65.5% vs. 64.9%, *p* = 0.666). The prevalence of SHS exposure among non-smokers was higher in those who worked 40–70 h per week, had shorter duration of stay, those whose employment was agricultural, unemployed or other, those with an employer within the system, and those who had migrated between counties.

### Knowledge of hazards of smoking

Migrants’ knowledge of hazards of smoking are shown in Table 3. About 95% of all participants were aware that lung cancer is one of the hazards of smoking and there was no difference between current smokers (94.7%) and non-smokers (95.0%). Nevertheless, more non-smokers had knowledge that fertility reduction is a hazard of smoking than current smokers (56.0% vs. 49.4%, *p* < 0.001). More non-smokers also had knowledge that accelerated aging is a hazard of smoking than current smokers (56.0% vs. 49.4%, *p* < 0.001). All participants’ knowledge of cardiovascular diseases (43.8%), heart disease (35.0%) and stroke (26.2%) as hazards of smoking were comparatively low compared with their knowledge of lung cancer (94.8%), fertility reduction (54.4%) and accelerated aging (53.8%).

### Multivariate analyses

Sex-stratified multivariate logistic regression results are shown in Table 4. In the male model, age (aOR = 1.02, 95% CI: 1.01–1.03), years of education (aOR = 0.95, 95% CI: 0.93–0.98), and duration of stay (aOR = 0.99, 95% CI: 0.97–1.00) were factors significantly associated with current smoking. Other factors included the type of occupation (business and service personnel (aOR = 1.25, 95% CI: 1.03–1.53); production, transportation and facility operation personnel (aOR = 1.52, 95% CI: 1.23–1.88)) and trans-county migration (aOR = 1.32, 95% CI: 1.05–1.66). Employment status, working hours per week, household registration status, and the nature of employment were not associated with current smoking among males.

**Table 4:** Logistic regression of general characteristics associated with smoking behavior

	Current smokers								SHS			
	Male				Female							
Predictors	aOR	Lower 95% CI	Upper 95% CI	*P*	aOR	Lower 95% CI	Upper 95% CI	*P*	aOR	Lower 95% CI	Upper 95% CI	*P*
Age	1.02	1.01	1.03	< 0.01	1.03	1.00	1.06	**0.05**	1.01	1.00	1.02	0.15
Education year	0.95	0.93	0.98	< 0.01	0.97	0.90	1.05	0.44	0.98	0.96	1.01	0.14
Duration of stay	0.99	0.97	1.00	0.01	0.98	0.95	1.02	0.40	0.99	0.98	1.00	0.03
Working hours per week	1.00	0.99	1.00	0.50	1.01	0.99	1.02	0.44	1.00	1.00	1.00	0.79
Marital status												
Never married	1.00	1.00	1.00		1.00	1.00	1.00		1.00	1.00	1.00	
Married	0.87	0.72	1.05	0.14	0.72	0.39	1.36	0.32	0.85	0.70	1.03	0.10
Divorced/widowed	1.16	0.56	2.38	0.69	3.20	1.00	10.25	0.05	0.48	0.25	0.91	0.02
*Household registration* status												
Agricultural	1.00	1.00	1.00		1.00	1.00	1.00		1.00	1.00	1.00	
Nonagricultural	0.92	0.78	1.08	0.31	1.70	1.03	2.79	0.04	1.07	0.91	1.27	0.41
Employment												
Yes	1.00	1.00	1.00		1.00	1.00	1.00		1.00	1.00	1.00	
No	1.12	0.96	1.31	0.15	1.25	0.75	2.08	0.39	0.99	0.84	1.15	0.86
Occupational type												
Professional and technical personnel	1.00	1.00	1.00		1.00	1.00	1.00		1.00	1.00	1.00	
Business and service personnel	1.25	1.03	1.53	0.03	0.67	0.34	1.35	0.26	1.29	1.05	1.58	0.02
Production, transportation and facility operation personnel	1.52	1.23	1.88	< 0.01	0.72	0.32	1.64	0.43	1.13	0.89	1.42	0.32
Agricultural, unemployed and others	1.14	0.73	1.79	0.55	0.28	0.07	1.11	0.07	1.58	1.10	2.26	0.01
Employer nature												
Within the system	1.00	1.00	1.00		1.00	1.00	1.00		1.00	1.00	1.00	
Outside the system	0.97	0.78	1.20	0.79	0.62	0.31	1.24	0.17	0.77	0.60	0.97	0.03
Agricultural, unemployed and others	1.03	0.76	1.40	0.84	0.60	0.22	1.68	0.33	0.54	0.39	0.75	< 0.01
Migration type												
Trans-provincial	1.00	1.00	1.00		1.00	1.00	1.00		1.00	1.00	1.00	
Trans-municipal	0.89	0.77	1.02	0.10	0.88	0.54	1.43	0.61	1.00	0.87	1.15	0.99
Trans-counties	1.33	1.06	1.66	0.02	1.36	0.70	2.64	0.36	1.54	1.20	1.98	< 0.01

In the female model, age (aOR = 1.03, 95% CI: 1.00–1.06), household registration status (aOR = 1.70, 95% CI: 1.04–2.80) and marital status (aOR = 3.20, 95% CI: 1.00–10.25) were the factors significantly associated with smoking. Employment status, though significantly associated with current smoking on the bivariate analysis, was no longer significant on the multivariate analysis.

The multivariate logistic regression results of determinants of SHS exposure among non-smokers are shown in Table 4. Duration of stay (aOR = 0.99, 95% CI: 0.98–1.00), marital status (aOR = 0.48, 95% CI: 0.25–0.91) and trans-county migration (aOR = 1.54, 95% CI: 1.20–1.98) were associated with SHS exposure. Other factors significant in the bivariate analysis, including working hours per week and employment status, were no longer significant in the logistic regression model.

## Discussion

This is the first large-scale study to investigate SHS exposure and knowledge of smoking hazards among internal migrants in China. The large difference in current smoking prevalence by sex is similar to previous studies [1, 13, 14, 25]. For male migrants, the prevalence of current smoking in this study (54.4%) was slightly higher than that for the general male population (52.9%) and for urban male residents (49.2%), but lower than for rural male residents (56.1%), as reported in the 2010 GATS [2]. For female migrants, the prevalence of current smoking in this study (3.7%) was higher than females living in both rural (2.2%) and urban (2.6%) settings, as reported in the 2010 GATS [2]. This study’s prevalence estimates are higher than nearly all other national and subnational estimates of smoking prevalence in China [1, 13, 14, 25] except one [26]. These variations in reported rates of smoking may be attributed to the different study locations, sampling frames and demographic characteristics of the populations enrolled.

Studies show that men may face increased restrictions on tobacco use at work or increased social pressure not to smoke, resulting in reduced smoking after migration, whilst women may have enhanced independence, less restrictive social norms and higher incomes, resulting in higher smoking rates after migration [14, 27]. Three previous studies [1, 13, 26] supported this trend while another two reported discrepancies [14, 25].

Lack of education can limit awareness of the hazards of smoking and can account for the higher prevalence of smoking among less educated persons [28], as was found in our and in other studies [14, 29]. Longer duration of stay may indicate better inclusion of migrants in their new areas and less social stress, and therefore less tobacco use, as was shown in our results, although the evidence on this is mixed [1, 14, 15]. Evidence for smoking prevalence among migrants by occupation also continues to be highly inconsistent [1, 15], and our analysis could add further insight to this debate. We found that male migrants who migrated between counties had a higher prevalence of smoking than those who migrated between provinces and between municipalities; no previous study examined this association in this population.

For female migrants, the logistic regression model showed non-agricultural female migrants had increased odds of current smoking than agricultural female migrants. In the study by Ji et al.*,* however, there was no significant association between household registration status and smoking in female migrants [14]. Our model showed that divorced/widowed females had 3.20 times the odds of current smoking compared to never married and married females, while in the study by Ji et al., again no such difference was found [14].

In this study, the prevalence of SHS exposure among non-smokers was 65.1%, which was lower than the national prevalence reported in the 2010 GATS [24] (72.4%) but similar to a study by Huang et al. (68.7%) [30]. Our determinants of SHS exposure are somewhat concordant with those of Huang et al., in that they too found occupation to be a significant determinant of SHS exposure, however they also found determinants that we did not, such as age and education [30].

Our results show that migrants’ knowledge of the hazard of ‘smoking-associated lung cancer’ was far greater than their knowledge of other hazards. This finding is consistent with a study by Finch et al. on rural-to-urban migrant women in Beijing [20], and a further national study [31]. Both studies reported that non-current smokers’ knowledge of smoking-associated accelerated aging was significantly higher than current smokers’ knowledge, indicating that health education on smoking hazards might help to control and reduce smoking prevalence.

To our knowledge, ours is only the second study that has analyzed a large-scale migrant sample for smoking prevalence and its determinants. Our results provide overall levels of migrant smoking prevalence and the influences of migration-related factors on smoking. This is also the first large-scale study to investigate SHS exposure among non-smokers and knowledge of smoking hazards among internal migrants. These results provide a knowledge base for developing or improving tobacco control interventions among migrants, such as focusing on divorced/widowed women.

This study is not without limitations, however. First, it is a cross-sectional study so we cannot draw conclusions regarding causality. Secondly, it used typical sampling methods rather than random sampling so the point estimates and associated variance estimates might be biased. Thirdly, as the data used were not obtained from a smoking-specific survey, the responses were limited smoking status, exposure to SHS and knowledge of the hazards of smoking. Lastly, weighting was not performed prior to the analysis, so there is a chance that the probability of selection of respondents was not equal.

## Conclusion

The prevalence of current smoking and SHS exposure among internal migrants were high. Risk factors associated with current smoking among male migrants include age, years of education, duration of stay in a location after migrating there, occupational type and migration type. Among female migrants risk factors include age, household-registration status and marital status. Factors associated with SHS exposure among non-smokers include duration of stay, marital status, occupation type, nature of employment and migration type.

Our findings suggest the need for specifically targeted tobacco control interventions for the rapidly growing and changing internal migrant population. We recommend further tobacco control measures to address the identified risk factors. Due to the limited number of studies and the inconsistencies between studies, further specialized research into the smoking behaviors of internal migrants in China is required in order to determine whether similar factors may contribute to smoking behavior nationwide.

## Abbreviation

SHS: Second-hand smoke
GATS: Global Adult Tobacco Survey

## Footnotes

1 Outside the system: refers to those who are not within the formal sector but are not unemployed and are not working in agriculture.

2 Within the system: refers to civil servants system and those employed by public institutions and state-owned enterprises.
